# A Non-Invasive Diagnostic Platform for Canine Leishmaniasis Using VOC Analysis and Distributed Veterinary Infrastructure

**DOI:** 10.3390/vetsci12080732

**Published:** 2025-08-04

**Authors:** Marius Iulian Mihailescu, Violeta Elena Simion, Alexandra Ursachi, Varanya Somaudon, Aylen Lisset Jaimes-Mogollón, Cristhian Manuel Durán Acevedo, Carlos Cuastumal, Laura-Madalina Lixandru, Xavier Llauradó, Nezha El Bari, Benachir Bouchikhi, Dhafer Laouini, Mohamed Fethi Diouani, Adam Borhan Eddine Bessou, Nazim Messaoudi, Fayçal Zeroual, Valentina Marascu

**Affiliations:** 1 Faculty of Engineering and Computer Science, SPIRU HARET University, 47 Fabricii Street, 076144 Bucharest, Romania; v.marascu.mi@spiruharet.ro; 2 Faculty of Veterinary Medicine, SPIRU HARET University, 256 Basarabia Boulevard, Sector 3, 030045 Bucharest, Romania; ushmv_simion.violeta@spiruharet.ro (V.E.S.); ursachi.alexandra.mv@gmail.com (A.U.); 3 MUI-ROBOTICS, Moo 1, Rattanathibet Rd., Saima, Nonthaburi 11000, Thailand; varanya.mui@gmail.com; 4 Faculty of Engineering and Architecture, University of Pamplona, Pamplona 543050, Colombia; lisset.jaimes@unipamplona.edu.co (A.L.J.-M.); cmduran@unipamplona.edu.co (C.M.D.A.); carlos.cuastumal@unipamplona.edu.co (C.C.); 5 Institute of Veterinary Medicine and Animal Sciences, Estonian University of Life Sciences, Kreutzwaldi 62, 51014 Tartu, Estonia; laura-madalina.lixandru@emu.ee; 6 R&D Digital Health Department, NVISION, 08028 Barcelona, Spain; xavier.llaurado@nvision.es; 7 Biosensors and Nanotechnology Group, Faculty of Sciences, Moulay Ismaïl University, Zitoune, Meknes B.P. 11201, Morocco; nezhaelbari6@gmail.com (N.E.B.); benachir.bouchikhi@gmail.com (B.B.); 8 Laboratoire de Micriobiologie Moléculaire, Vaccinologie et Biotechnologie Appliquée/LR16IPT01, Institut Pasteur de Tunis, Université Tunis Elmanar, Tunis 1068, Tunisiafethi.diouani@gmail.com (M.F.D.); 9 Chadli Bendjedid University, El-Taref, BP N° 73, EL Tarf 36000, Algeria; a.bessou@univ-eltarf.dz (A.B.E.B.); n.messaoudi@univ-eltarf.dz (N.M.); zeroual-faycal@univ-eltarf.dz (F.Z.); 10 National Institute for Laser, Plasma and Radiation Physics, 409 Atomistilor Street, 077125 Magurele, Romania

**Keywords:** machine learning, non-invasive testing, cloud-based architecture, artificial intelligence in animal health, veterinary diagnostics, canine leishmaniasis, volatile organic compounds (VOCs)

## Abstract

This article describes a new software architecture for the non-invasive detection of canine leishmaniasis disease. The proposed platform combines gas-sensing technologies, artificial intelligence (AI), and modular cloud-based software components to identify disease-specific volatile organic compounds (VOCs) found in dog breath and hair samples. The system, which has a multi-tier architecture that includes data collection, pre-processing, machine learning-based analysis, diagnosis-request processing, and user interfaces for veterinarians, faculty researchers, and dog owners, has been integrated into a Li-ion Power website plug-in. The primary goal of implementing the proposed platform is to detect parasites at any point they are infectious to a host. This includes detecting parasites at all stages of their life cycle, where they can infect a new host. In addition, this is crucial for accurate diagnosis, effective treatment, and preventing further transmission.

## Simple Summary

Canine leishmaniasis is a serious disease that affects dogs and can also impact humans. Traditional ways to detect this disease involve taking blood or tissue samples, which can be stressful for the animals and require specialized equipment. In this paper, we developed a new, non-invasive way to detect leishmaniasis in dogs by analyzing special chemicals released in their breath and hair. Using smart sensors and artificial intelligence, our system can recognize these chemical patterns that are linked to infection. We also created a cloud-based platform that connects veterinary clinics, hospitals, universities, and pet owners, making it easy to collect and share test results. The system was tested on samples from 72 dogs and proved to be accurate and reliable. This approach could make disease detection faster, less painful, and more accessible, especially in areas with fewer veterinary resources. It may also help in controlling the spread of the disease and protecting both animal and human health. Our solution is designed to be expanded in the future to detect other diseases and support better care for pets worldwide.

## 1. Introduction

Canine leishmaniasis is a significant zoonotic disease caused by protozoan parasites of the genus *Leishmania*, transmitted primarily through the bites of infected female sandflies [1]. It is endemic in areas of southern Europe; the Mediterranean basin; and certain areas of Africa, Asia, and South America. In dogs, the disease may present as a chronic, systemic infection and can result in severe dermatological, renal, and ocular complications if not treated [2,3]. Canine leishmaniasis is classified as a neglected tropical disease (NTD) with a significant impact on human and animal health, particularly in impoverished communities [4]. The emergent trend of moving beyond sporadic, localized rural infections towards mass spread in chaotic, rapidly urbanizing areas, with dogs serving as potential reservoirs of disease, underlines the considerable socio-economic impact of this disease globally. Another important aspect is the spread of canine leishmaniasis caused by *Leishmania infantum* (*L. infantum*) in urban areas and peri-urban areas [5].

Standard diagnostic methodologies are based on invasive sampling techniques, e.g., blood draws, lymph node or bone marrow aspirates, and subsequent serological or molecular testing. Although effective, these methods are limited by their high operating costs, specialized equipment, the need for trained personnel, and the physical distress experienced by animals. Such limitations stifle access to early diagnosis, especially in resource-limited or rural veterinary settings where disease burden is often greatest.

There is a growing need for alternatives to diagnostics that represent a more accessible [6], animal-friendly [7], and scalable option [8]. In addition, recent advances both in volatile organic compound (VOC) sensing technologies [9] and machine learning have opened new frontiers in non-invasive diagnostics [10]. VOCs are low molecular weight metabolites emitted through biological processes, and some VOC signatures have been linked to infectious or metabolic conditions, including parasitic diseases, such as leishmaniasis [11,12].

This paper proposes a new, software-based diagnostic system to assist with non-invasive diagnosis of canine leishmaniasis using VOCs sampled from breath and hair samples. The platform provides automated, real-time diagnostic capabilities by combining gas-sensing devices, AI/ML models, and cloud-based software infrastructure. Moreover, the system links a range of stakeholder groups (e.g., veterinary clinics, hospitals, research faculties, and dog owners) across distributed networks, facilitating information sharing and care coordination. This paper is mainly aimed at designing a software architecture for data capturing, processing, and VOC analysis. By utilizing AI models, the platform helps predict future leishmaniasis infections. Also, the proposed platform is secure and provides scalable access between multiple veterinary and public stakeholders, along with enabling early, accessible, and affordable diagnostic testing for more dogs.

This paper presents the design, implementation, and validation of a modular, AI-augmented diagnostic platform to assist veterinary practitioners and dog owners in making swift and accurate decisions concerning canine health and disease management, enabling the practice of preventive veterinary care.

## 2. Related Works

### 2.1. Traditional Diagnostic Techniques for Canine Leishmaniasis

Canine leishmaniasis has traditionally been diagnosed through invasive clinical procedures, including cytology; histopathology; and molecular testing, such as polymerase chain reaction (PCR). Serological methods, such as enzyme-linked immunosorbent assays (ELISAs), immunofluorescence antibody tests (IFATs), and direct agglutination tests (DATs), are commonly used to detect anti-*Leishmania* antibodies [13,14]. However, their reliance on trained personnel, well-equipped laboratories, and stress-inducing sample collection restricts their use in decentralized or field-based environments [15].

### 2.2. Non-Invasive Diagnostics and VOC Analysis

As humane, scalable, and rapid alternatives, non-invasive diagnostic techniques are being studied. The examination of VOCs emitted from biological samples, such as breath, skin, hair, urine, or feces, has demonstrated potential in the diagnosis of diseases in both humans and animals [16]. In human medicine, VOC profiling is applied to detect cancers, infectious diseases, and metabolic disorders [17,18]. Related studies in veterinary research have shown that certain VOCs are correlated with parasitic or bacterial infections, including gastrointestinal pathogens and vector-borne diseases in animals. Various methods, such as gas chromatography–mass spectrometry (GC-MS) and electronic noses, have been studied to measure and characterize VOC patterns in previous research [2].

Epidemiological evidence for the detection of leishmaniasis by VOCs of the breath or hair is found in work showing significant differences in the VOC profiles between infected and non-infected dogs [19].

### 2.3. Artificial Intelligence and Machine Learning in Veterinary Diagnostics

With applications from image-based disease classification and behavioral analysis to predictive diagnostics using electronic health records, Artificial Intelligence (AI) and Machine Learning (ML) techniques have quickly permeated veterinary medicine [20,21]. Supervised learning algorithms (e.g., decision trees, support vector machines [SVMs], and random forests [22,23]) are frequently used to predict medical conditions from structured or unstructured data. To improve the detection of infectious diseases and animal predictive accuracy, AI has been combined with biosensing technologies in various applications for both livestock and pets. Yet, the application of AI-assisted diagnostics for canine leishmaniasis is lacking.

### 2.4. Veterinary Health Platforms and Distributed Systems

Today’s veterinary platforms primarily cover areas such as Electronic Health Records (EHRs), appointment scheduling, teleconsultation, and inventory management. These may include cloud-based systems, such as eVetPractice [24], Provet Cloud, and VetCompass [25], which together provide a complementary, centralized solution for clinic operations. However, these platforms have no integration with diagnostic hardware, AI modules, or in-the-moment sample analysis systems. Herein, they are primarily intended for use in local clinics, rather than for working across multiple veterinary hospitals and faculties and with remote pet owners.

## 3. Methodology

This section outlines the methodological framework for the non-invasive diagnosis of canine leishmaniasis using VOC analysis. It encompasses standardized sample collection, VOC data acquisition, pre-processing, and integration with the AI-enhanced diagnostic pipeline.

The process of VOC extraction and pre-processing involves configuring a gas-sensing device, where VOC analysis is carried out using portable gas-sensing arrays (“e-noses”) with Multi-Walled Carbon Nanotube (MWCNT)/polymer sensors. Each device includes four VOC-specific sensors, calibrated for the detection of aldehydes, ketones, alkanes, and alcohols, which are connected to metabolic changes in leishmaniasis. Additionally, it features inbuilt filters to prevent contamination and Wi-Fi modules for the real-time transfer of raw sensor signals to a centralized processing unit. The *data acquisition pipeline* includes the following:

During *sensor signal acquisition*, VOC sensors are used to continuously characterize the concentration change of the compound and generate analog voltage signals. During *signal conversion*, every analog signal is converted to digits through an onboard Analog-to-Digital Converter (ADC) and is also time-stamped. Also, in *transmission*, data packets are encrypted and sent to the cloud service through a secure Representational State Transfer Application Programming Interface (RESTful API), which is an architectural style for designing networked applications with a set of rules that allows different software systems to talk to each other. Inside *storage*, the VOC signals, accompanied by metadata and labels (CONTROL/INFECTED), are stored in the platform’s VOC Dataset Storage module.

The following pre-processing techniques are applied to improve the signal quality and help the models learn better:

In *baseline correction*, the drift of the sensor over time is corrected based on the subtraction of a moving baseline signal inferred from clean air reference data. Through The noise reduction process, low-pass filters, as well as smoothing algorithms (such as the Savitzky–Golay filter) can be used to reduce environmental and electrical noise. Applying the *normalization method*, the sensor values are normalized across different devices using Z-scores or min–max scaling to accommodate variability in device sensitivity. Additionally, feature extraction was used to extract statistical (mean, variance, and skewness), frequency-domain (FFT), and temporal response (including rising time and fall time) features from the VOC curves. Last, but not least, by using *label mapping*, each sample is annotated with its corresponding clinical classification (CONTROL/INFECTED) for supervised learning. The output of this pipeline is a feature vector for each sample, which is eventually used for AI-based classification models, as explained in Section 4.

## 4. AI and Machine Learning Models

The VOC-based diagnostic system relies on supervised ML techniques to classify canine samples as INFECTED or NON-INFECTED (CONTROL) based on their volatile organic compound signatures. This section details the feature selection strategy, training procedures, and evaluation of predictive performance.

### 4.1. Feature Selection

The fitness and distinguishing capacity of input features are essential for the performance of ML models. A set of 6 features is extracted from each VOC sample. First, the *banking statistical descriptors* consist of the mean, standard deviation, skewness, and kurtosis. Further on, the *temporal features* consist of rise-response/recovery and peak width time. Moreover, the *signal frequency components* are obtained using the Fast Fourier Transform (FFT). Herein, derivative-based features are used, such as slope and inflection points.

The initial feature importance can be derived from domain knowledge, correlation heatmaps, and univariate statistical tests (such as ANOVA and *t*-tests) comparing CONTROL and INFECTED samples. Features with low variance or multicollinearity are eliminated, thereby reducing overfitting.

The identified predictors were ranked by implementing recursive feature elimination (RFE) and tree-based importance scores (using Random Forest classifiers), selecting the best-performing subset for use in the classification tasks.

### 4.2. Model Training

*Dataset construction.* The training set includes labeled samples (CONTROL or INFECTED) from distributed clinics, hospitals, and veterinary faculties. Each computer/entry consists of a VOC feature vector along with metadata. As a result, SMOTE (*synthetic minority over-sampling technique*) is applied if the distributions are highly skewed to balance the classes.

*Modeling techniques*. Several supervised learning models are implemented and compared, including a *Random Forest Classifier* (*RFC*), which is resistant to noise and is effective with limited data. The 4-Support Vector Machine (SVM) has a radial basis function (RBF) kernel. Gradient Boosting Machines (GBMs) for tabular VOC data are optimized. Additionally, Multilayer Perceptron (MLP) is utilized for learning about nonlinear feature interactions. Each model was trained using stratified 10-fold cross-validation. To optimize hyperparameters, we employ a grid search with cross-validation.

*Evaluation of metrics.* We used standard classification metrics to evaluate the models, such as accuracy, which is the ratio of correct predictions made by the model. *Precision* and recall are crucial, especially for identifying disease positives. The F1 Score is the harmonic mean between precision and recall. Additionally, the AUC-ROC Curve is used to evaluate the model’s ability to separate classes across various thresholds.

### 4.3. Results and Analysis

*Feature importance.* The most significant VOC features included are the peak amplitude of VOCs 2 and 3 from the volatile gases, the rise time of VOC 1, and signal skewness in VOC 4.

Most importantly, these features were consistently ranked across their respective models, validating their biological relevance in leishmaniasis progression.

*Error analysis.* Such false negatives were typically observed in samples with a low Voltage Input (VI)/Signal Input (SI) signal amplitude, approaching detection thresholds, or suspected co-infections. The sporadic non-infection-linked false positives reported during fights helped isolate recent antibiotic therapy as a cause, altering the metabolic profile of some non-infected dogs.

A separate validation set located away from the original model generation area tested model generalizability by demonstrating that the model’s performance would be consistent across multiple veterinarian locations, indicating if these models are resilient to environmental variability.

## 5. Data Analysis and Dataset Description

This section gives an overview of the dataset used during the pilot implementation phase of the proposed platform, followed by a preliminary analysis and interpretation of the volatile organic compound (VOC) signals collected. The data represent samples collected from canine subjects via breath and hair during field trials in veterinary clinics and hospitals.

### 5.1. Dataset Composition

The dataset contains 186 VOCs collected from 72 dogs, representing breath and hair samples. The initial dataset exhibited an imbalance, with approximately 25% infected cases (47 samples) and 75% control cases (139 samples), resulting in an approximate ratio of 1:3 infected-to-control cases. The synthetic minority over-sampling technique (SMOTE) was applied to balance this distribution, achieving a 1:1 ratio to ensure optimal model training. Every sample is annotated with the metadata: Sample ID, Sample Type (Breath/Hair), Tunisia Region (North, South, East, and West), Diagnosis Label (Infected/Control), VOC Sensor Signal (time series of voltage outputs), and Metadata for the collection (clinic ID, date/time, and environmental settings)

To prepare for analysis, each VOC signal was pre-processed and downsampled to a vector of engineered features: signal mean, maximum, minimum, skewness, rise time, and peak width.

### 5.2. Pre-Processing and Feature Engineering

As obtained, the vacuumed VOC sensor signals demonstrated typical noise patterns, affected by both the noises from ambient air and sensor drift (*example execution*, where baseline correction was performed using adaptive moving average filters; Savitzky–Golay filtering, which was used for signal denoising; *normalization* using min–max scaling with bounds specific to each sensor; and *feature extraction* for creating 20 statistical and temporal features for each sample). The structured dataset, in its final form, contained 20 numerical features and one categorical output label that was used as input for machine learning.

### 5.3. Exploratory Data Analysis (EDA)

**Figure 1:**
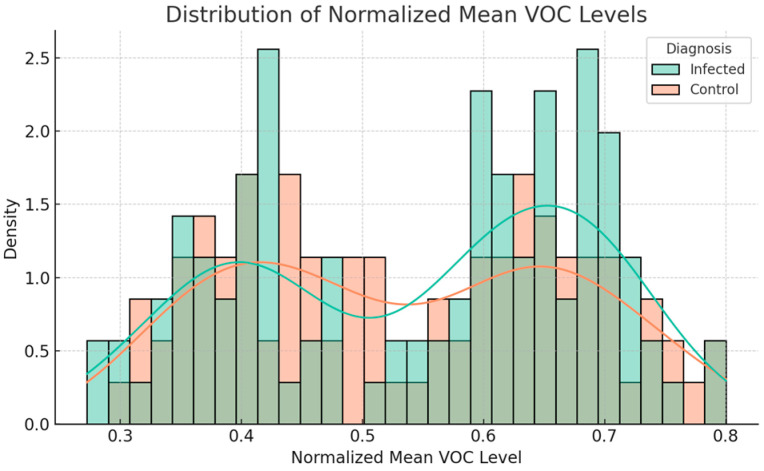
Distribution of normalized mean VOC levels between INFECTED cases and CONTROL cases.

The initial exploration of the data indicated relevant differences in VOC signals between INFECTED and CONTROL samples. A statistically significant difference in normalized average VOC levels was found between INFECTED cases and CONTROLS. The normalized data were tested using the Shapiro–Wilk test, and equality of variances was assessed using Levene’s test. Inter-group comparisons of normalized mean VOC levels (INFECTED vs. CONTROL) were performed using Student’s independent samples *t*-tests. The appropriateness was further validated to assess the robustness of the proposal using the non-parametric Mann–Whitney U test. Cohen’s d and rank–biserial correlation were used to estimate effect sizes. The level of significance was set at α < 0.05. The tests indicated that the data distributions were not highly different from normal (Shapiro–Wilk INFECTED: W = 0.98, *p* = 0.65; CONTROL: W = 1.00, *p* = 0.97). Levene’s test showed no variance difference (F = 0.45, *p* = 0.50). An independent samples *t*-test exposed a difference in mean normalized VOC levels between INFECTED (M = 0.64, SD = 0.08) and CONTROL DOGS (M = 0.41, SD = 0.07), *t*(184) = 18.14, *p* < 0.001, *d* = 3.06). The Mann–Whitney U test supported the results (U = 6442.0, *p* < 0.001, rank–biserial correlation = –0.97), underscoring the stability of the results in light of the bimodal aspect in Figure 1. While the histograms in Figure 1 are shown to be bimodal, testing for normality as well as for homogeneity of variances resulted in an approximate normalization and homogeneity of variances, allowing for parametric analysis. This bimodality may represent biological diversity of the dogs, e.g., differences in parasite load, immune response, or the environment. The significant difference between the INFECTED and CONTROL groups persisted, both numerically and statistically, throughout both parametric and non-parametric analyses, indicating its robustness.

The mean normalized VOC level distributions of the two classes are presented in Figure 1.

Supplementary regional breakdowns of sample labels also demonstrated that the northern and western clinics had low incidence rates of positive cases, consistent with known patterns of vector activity for the MA ICTs (*Information and Communications Technology in the context of Massachusetts*). These trends were visualized using stacked bar plots on the epidemiology page from the dashboard, which could support academic research as well as clinicians.

**Figure 2:**
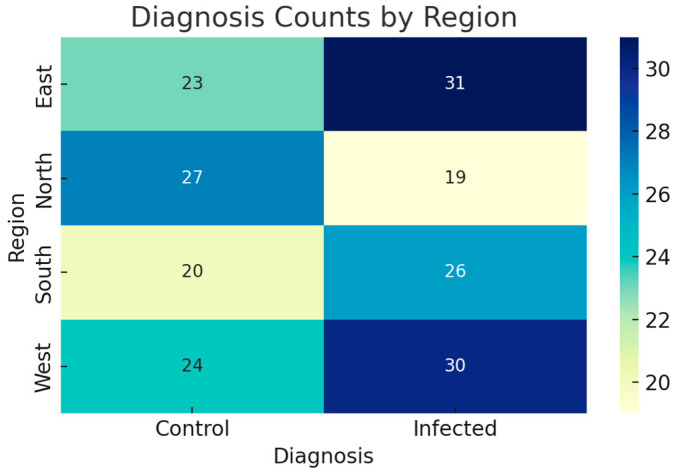
Diagnosis counts by region: used for estimating the spatial distribution of positive diagnoses, examining the regional (from Tunisia) infection rates.

To estimate the spatial distribution of positive diagnoses, we examined the regional infection rates. Figure 2 represents a heatmap of diagnosis counts for regions with high infection rates in the South and East regions (from Tunisia), indicating geographic clustering or environmental correlation, as sandflies thrive in warmer and more humid areas.

### 5.4. Interpretation of Key Signal Features

A few of the features stood out as significant predictors of infection and were confirmed both statistically and by model feature importance scores. In particular, the breath sensors demonstrate high discriminative power with respect to peak amplitude. Further, the time to maximum signal (rise time) was significantly shorter in infected dogs. Asymmetric VOC emission patterns, in terms of signal variance and skewness, were observed, which may accompany metabolic alterations due to the parasitic infection. The proposed approaches can be considered a useful tool for future evidence demonstrating that *L. infantum* is capable of stealthily modulating host metabolism in a detectable fashion (ultimately observable via volatile excretions), supporting the biological hypothesis therein. However, in this paper, we will not focus on this subject.

**Figure 3:**
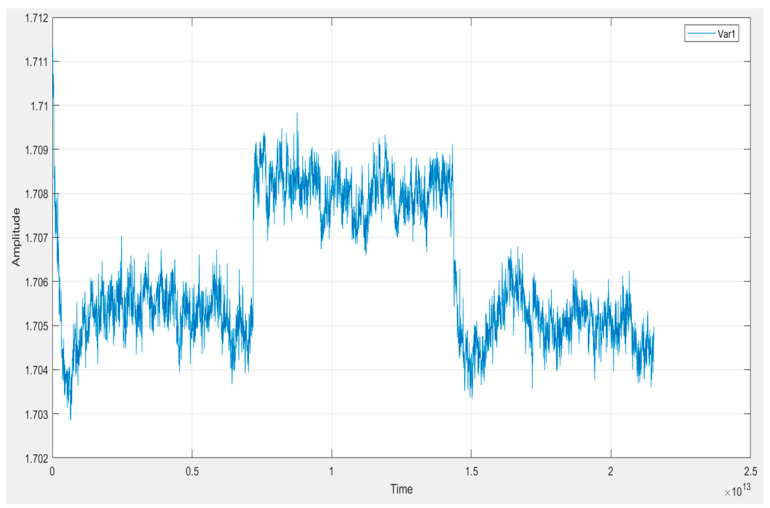
FFT plot of the Averaged Spectrum of the signal’s characteristics: its frequency components, time-varying behavior, and the presence of peaks or significant events.

In Figure 3, we observe an example of an extracted signal from our first dog sample. This investigation helps us to understand the signal’s characteristics, including its frequency components, time-varying behavior, and the presence of peaks or significant events.

**Figure 4:**
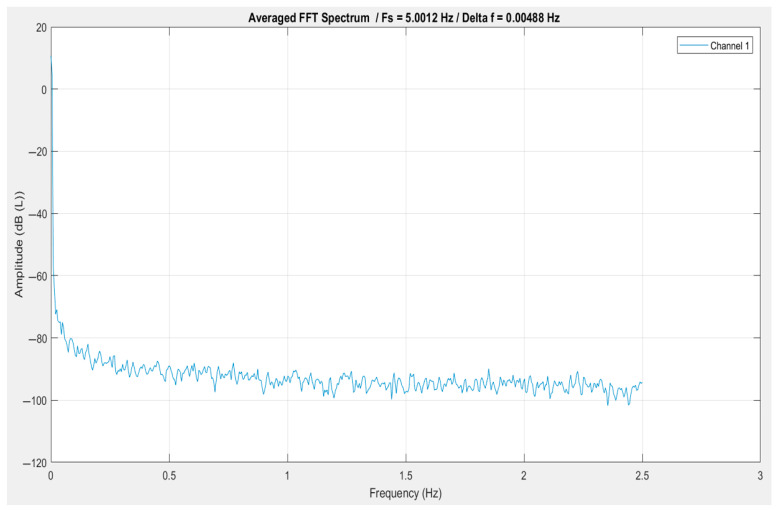
FFT Plot of Averaged Spectrum: a frequency domain representation of the signal obtained through the average FFT method.

The frequency domain representation of the signal, obtained through the average FFT method (see Figure 4), reveals that the signal contains dominant low-frequency content, with a steep drop in amplitude observed at frequencies approaching 0 Hz. This is a strong indicator of quasi-static or slowly varying signal behavior, possibly including baseline drift or low-frequency physiological phenomena.

The lack of sharp spectral peaks throughout the frequency range up to approximately 2.5 Hz suggests an absence of strong periodic or oscillatory components. Instead, the signal appears to be broadband and noise-like, with a generally flat spectrum at lower amplitudes beyond the initial decline. The noise floor remains stable at around −90 to −100 dB, indicating that the system’s noise is relatively uniform and not dominated by any specific frequency band.

The fact that this spectrum was averaged further validates that these low-frequency components are consistent across multiple data segments and not the result of transient noise.

**Figure 5:**
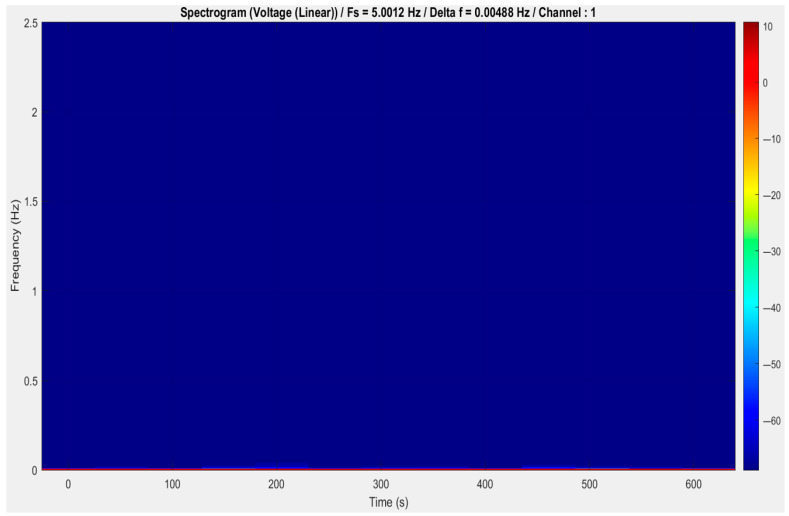
Time–frequency analysis: data sample spectrogram. Herein, the amplitude in both time and frequency domains is relatively low and constant.

As can be seen in the spectrogram (see Figure 5), the amplitude in both the time and frequency domains is relatively low and constant. Here, the completely dark blue with no bright colored streaks or color variation implies that the signal has very small energy across the entire frequency spectrum and time duration.

Over time, no specific frequency bands can be detected, indicating that the signal does not exhibit well-defined periodic or transient characteristics.

There is uniform low-level energy across the spectrum, from 0 to 2.5 Hz—which corroborates the earlier observation in the FFT spectrum where no peaks were present.

Since the color does not change significantly, the input signal is considered stationary within the 10 min time window examined.

This might suggest that the signal is primarily just noise, or it contains low-frequency elements that are incredibly weak—and may be too weak to detect in a linearly scaled spectrogram. The profile, however, might be used under stable conditions, environmental sensors, and unstimulated or idle sensors. There is little to no measurable signal variation when systems are in standby mode.

Figure 5 shows the spectrogram of the analyzed signal, which does not exhibit any significant frequency or amplitude change as a function of time. During the entire observation period, the signal remains weak and constant. In combination with the FFT spectrum, this indicates that the signal is either *quantization noise,* which has no frequency component with any physical significance, or the *quasi-static process* with little oscillation.

This is expected behavior; if so, it means a healthy, stable system. Conversely, if dynamic behavior or responses were expected (e.g., within a physiological or event-driven context), this finding could indicate insufficient stimulation or a sensor fault.

**Figure 6:**
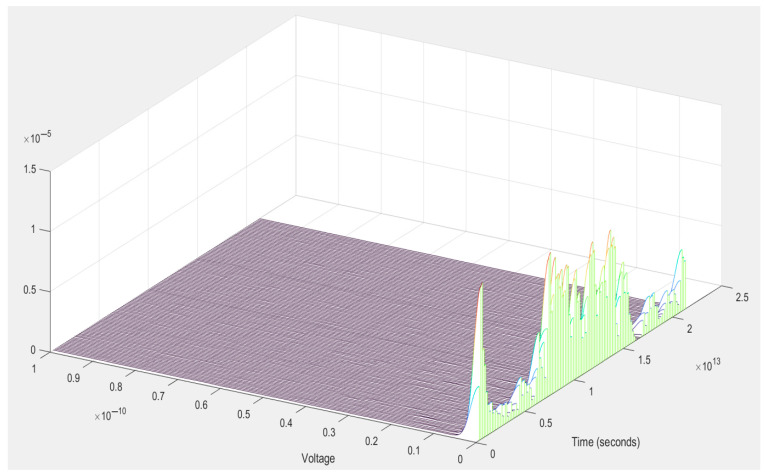
Plot of the detrended signal against time: the residual variation in a signal after detrending by subtracting linear or low-frequency trends.

The plot from Figure 6 shows the residual variation in a signal after detrending by subtracting linear or low-frequency trends, which helps reveal more subtle changes.

In Figure 6, the surface is mostly flat and near zero, indicating that most of the signal remained quiet or had low energy after detrending. In the latter quarter of our time axis, we notice several peaks in amplitude emerging from the surface. These appear as green/blue/orange ridges, indicating bursts or fluctuations in the detrended signal. Further, high activity in a small time frame indicates that an event or anomaly must have occurred within that window, whether it be a transient reaction to a stimulus or perhaps an artifact.

Signal characteristics can be summarized and interpreted as follows: the primary signal is incredibly faint (e.g., bioelectric signals or environmental information), it was an aggressive method of detrending, and the data contained a lot of meaningful variation that was removed. The neat, flat baseline over most of the timeline points indicates minimal or no activity outside of the burst region.

Post-detrending, this 3D plot reflects a mostly static signal and a short burst of activity over a specific time window. The spikes are the underlying dynamics that long-term trends have obscured.

**Figure 7:**
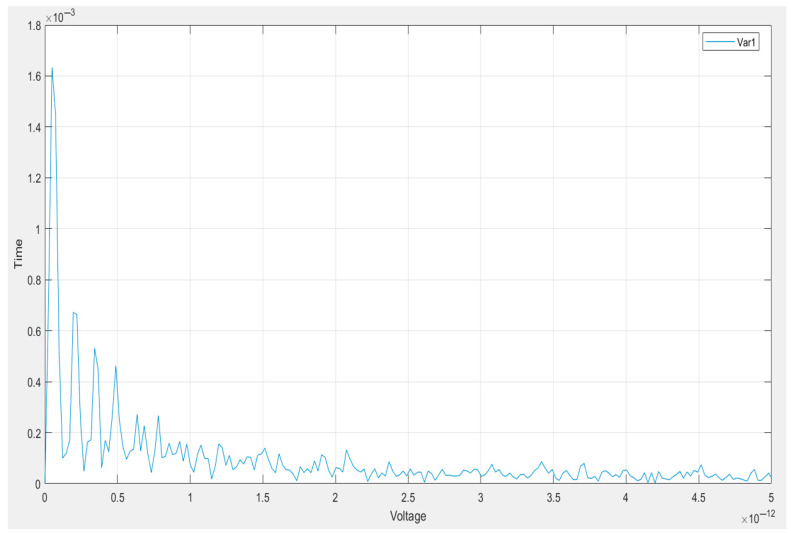
Plot of the voltage signal against time: the behavior of a voltage signal over time, which provides unique insight.

In Figure 7, we can see the behavior of a voltage signal over time, which provides unique insight. In the beginning, the signal shows a pattern of sharp spikes, indicating a phalanx of high-energy events in the very early minutes. This early peak indicates a sudden event or stimulus, followed by a rapid signal decay.

With higher voltages, the time values decrease rapidly and start to level out. This is the transition phase, during which the system settles into a low-activity steady state. The final part of the plot is flat, with little variation; this indicates that whatever energy was present in the system rapidly dissipated and that there was no significant subsequent dynamic behavior.

Such a response is typical for a “damped” system—one whose response to an initial disturbance returns to equilibrium without continuing oscillations. It is similar to the behavior observed in electrical devices, such as a discharging capacitor, or in mechanical systems undergoing damped vibrations. Similar profiles can also appear in biological recordings, where a physiological response occurs, followed by calm or recovery, or sensor readings capturing a temporary event.

One interesting point is the low voltage level, which is only in the order of picovolt (×10^−12^ V), indicating the high sensitivity of the system or sensor used. The signal’s tiny amplitude supports the theory that we are witnessing either a weak physical phenomenon or a highly sensitive detection process.

Overall, the voltage readout versus time shows a transient event with a short duration of activity, followed by a decaying signal that returns to an almost zero value. This behavior aligns with the previous analyses (i.e., FFT and spectrogram), which showed that no sustained or periodic signal components were present. Whether this pattern constitutes an accurate system reflection or an outlier of the experiment is a question of the larger experimental context.

**Figure 8:**
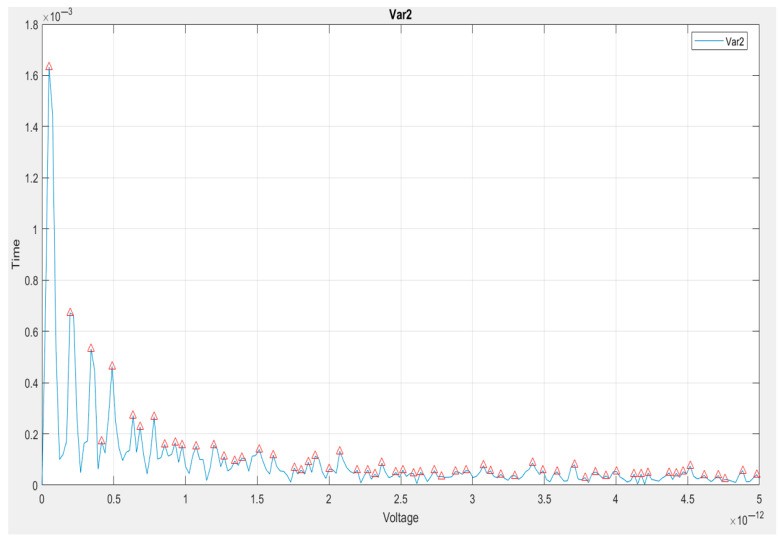
Plot of the FFT spectrum with the identified peaks and with the maximum energies in the frequency domain, which are the most dominant periodic components in the signal.

A peak (see Figure 8) is observed in the frequency domain and can be seen in the window’s FFT spectrum, which we plotted above. Accompanying these are also peaks in the local area and within the overall windowed Fourier Transform Spectrum. This takes us to the next level of spectral analysis, shifting from a view of the entire wolf pack to a focus on the most important members of the pack—the peaks.

Voltage (×10^−12^ V) is plotted on the x-axis and Time (again, potentially actually some representation of spectral amplitude or magnitude not explicitly time as the axis might indicate), on the y-axis. The second plot is named Var2, presumably a second variable or a processed version of a signal (maybe a filtered or transformed result).

*Peak identification*. On the FFT curve, small red triangles indicate the local maxima—the spectral peaks. These are the peaks with the maximum energies in the frequency domain, which are the most dominant periodic components in the signal. Herein, the peak is the highest one, at the lowest range of the applied voltage rates, so there is clearly a strong low-frequency component to the signal, or else, a DC offset.

This was followed by several small peaks at successively higher voltage values, whose frequencies became progressively smaller in amplitude. This indicated that the signal primarily contains low-frequency components and very weak contributions from higher-frequency waveforms.

At larger voltages, approximately ~1 × 10^−12^ V, the FFT spectrum greatly flattens, and the previously identified peaks become indistinguishable from the noise floor (which suggests little to no spectral relevance in this region).

*Interpretation of the signal*. This spectrum corroborates previous observations made through other plots (FFT, spectrogram, and time-domain analysis): the analyzed signal exhibits low-frequency content, in this case, with only a handful of components identifying it, and is not responsible for significant periodic hysteresis in the high-frequency content.

The red peak markers provide analytical clarity, useful for performing filtering or reconstruction on isolated components, for system resonances. Additionally, they are useful for anomaly detection in physical behaviors, particularly in identifying low-frequency periodicities.

### 5.5. Data Quality and Limitations

Despite overall data integrity, some limitations were observed, as sensor saturation occurred in 3% of hair samples due to high humidity. The inconsistent environmental metadata in 9% of entries is due to manual entry errors. Additionally, some regions were under-represented, which could potentially bias geographic models.

These limitations were addressed through data augmentation, the synthetic minority over-sampling technique (SMOTE), and model stratification techniques, as discussed in Section 4.

## 6. Software Architecture and System Design

The proposed system for non-invasive leishmaniasis diagnosis integrates gas-sensing hardware, artificial intelligence modules, user interfaces, and secure data pipelines across geographically distributed stakeholders. This section describes the layered software architecture, cloud-based infrastructure, geographic integration mechanisms, and security models that ensure compliance with veterinary health and data protection standards.

### 6.1. Multi-Tiered System Architecture

**Figure 9:**
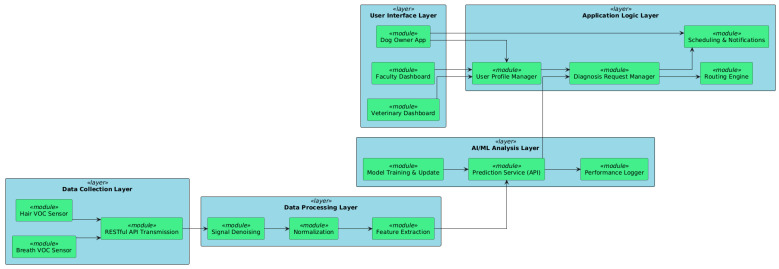
The multi-tiered system architecture developed in order to have a clear view of the dependencies between the user interface layer, application logic layer, AI/ML analysis layer, data processing layer, and the data collection layer.

Our system features a semi-decentralized, multi-tier, modular architecture (see Figure 9) which separates core responsibilities into distinct layers, allowing for better maintainability, scalability, and interoperability. The *data collection layer* interacts directly with external VOC sensors placed at veterinary practices and hospitals. Standardized hardware is used to collect data from breath and hair samples and transmit it via secure RESTful APIs to the backend. Further on, the *data processing and pre-processing layer* converts raw sensor signals through a pre-processing pipeline. It also engages in signal denoising, normalization, and feature extraction services. These operations are containerized and can be scaled out. The *AI/ML analysis layer* represents the trained machine learning models hosted (described in Section 4). It provides prediction services, such as APIs, which return probable diagnoses (INFECTED/CONTROL) and confidence scores. This layer also conducts model retraining and performance logging. Also, the *application logic* layer handles diagnosis requests, sample metadata, user profiles, and interaction rules between different stakeholders (vets, faculty, and dog owners). This layer comprises scheduling services, notification systems, and routing engines for coordination between clinics and hospitals. *Examples of the UI layer* include web-based dashboards for veterinarians, veterinary faculty, and dog owners, as well as mobile apps for veterinarians and dog owners. Based on the role of users, access is granted to relevant data and functions, such as uploading samples, reviewing a diagnosis history, or managing appointments.

This layered architecture enables us to integrate the new device data collection hardware into the loop for any user, facilitate cloud-based analysis, and support user interaction, thereby forming a comprehensive diagnostic pipeline with minimal effort.

### 6.2. Cloud Infrastructure

**Figure 10:**
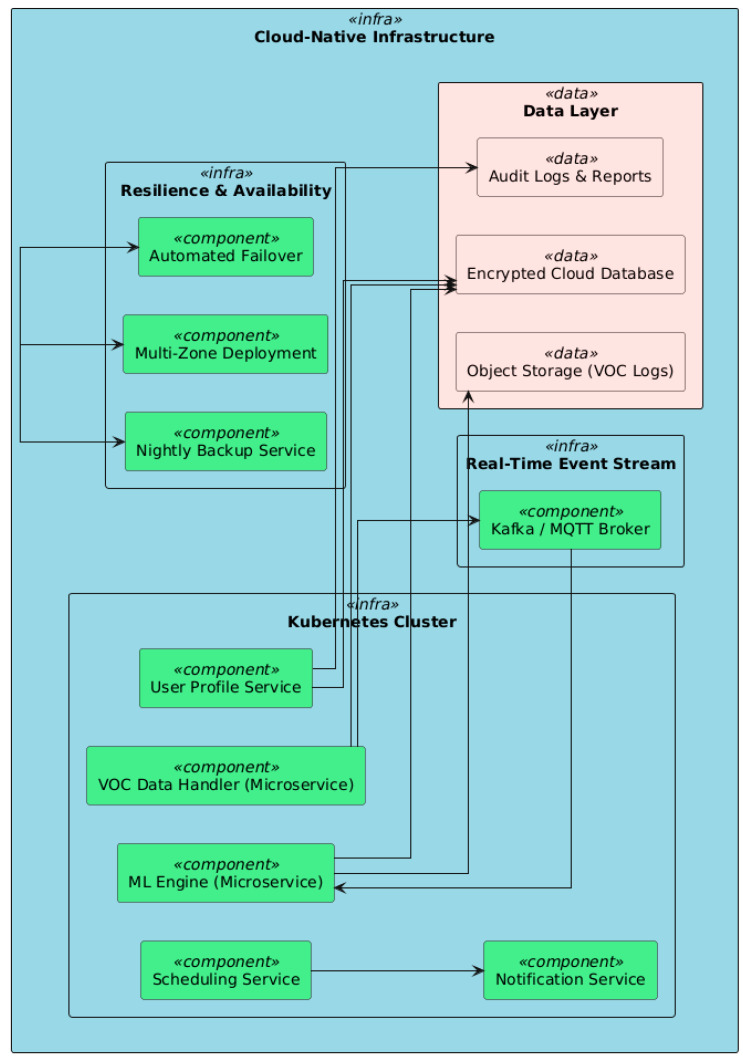
The cloud-native infrastructure ensures redundancy and scalability across sites and institutions.

The system is hosted on cloud-native architecture [26,27,28,29,30,31,32,33,34,35] (see Figure 10), ensuring redundancy and scalability across sites and institutions. Herein, we can identify the *containerized microservices*, where each core service (data handler, ML engine, scheduling, etc.) is encapsulated as an independent microservice managed by the Kubernetes cluster [36]. This allows load-balancing, resource allocation, and failure isolation. The *VOC data*, *user profiles*, *audit logs*, *and diagnostic reports* are stored in encrypted cloud databases. Raw data—e.g., sensor logs in native files—are stored in object storage with lifecycle management policies. Moreover, *time-critical monitoring* represents an architecture based on event handling (as in Kafka or MQTT), employed to implement the real-time processing of incoming sensor data, allowing near-instant feedback for diagnostics in the clinical environment. Also, by *high availability and redundancy*, the infrastructure is duplicated across availability zones, and automatic failover and nightly backups are in place to provide continuous service delivery.

### 6.3. Geographic Integration

**Figure 11:**
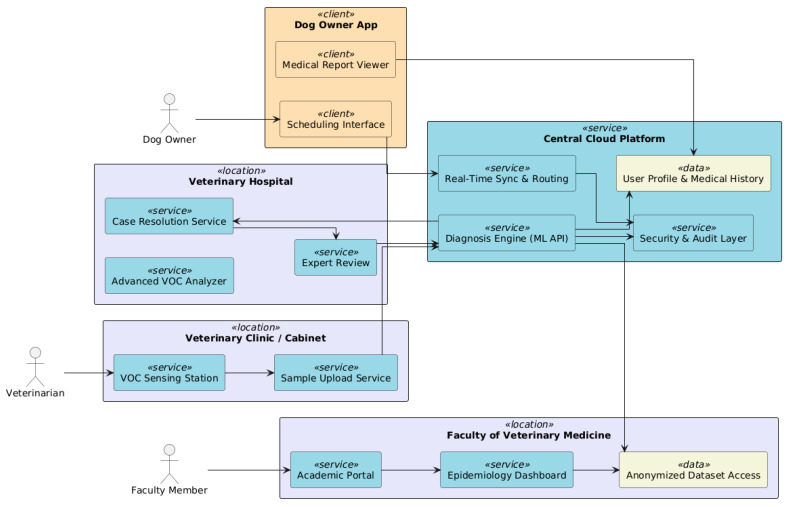
Geographic integration: secure, authenticated network unites all the locations, supporting real-time data, results, and decision workflow synchronization.

Fostering collaboration among geographically dispersed (see Figure 11) stakeholders holding enterprises, and Total Case Management (TCM) patient records is among the key design goals. In this line, *veterinary clinics and laboratories are the initial diagnostic* points where breath and hair samples are collected and sent. On the platform, each clinic is linked to a VOC sensing station. *Regional centers* are the more qualified centers for this activity, with suitable means and trained personnel. They receive and review complicated cases or undiagnosed diagnoses referred to them by clinics. The *Faculty of Veterinary Medicine* integrates academic input, takes part in case reviews, and utilizes anonymous data for research and epidemiological tracking. Also, the *dog owners* book diagnostics, see their dog’s medical history, and receive notifications via the mobile/web application, facilitating a secure, authenticated network that unites all the locations, supporting real-time data, results, and decision workflow synchronization.

### 6.4. Security and Privacy

**Figure 12:**
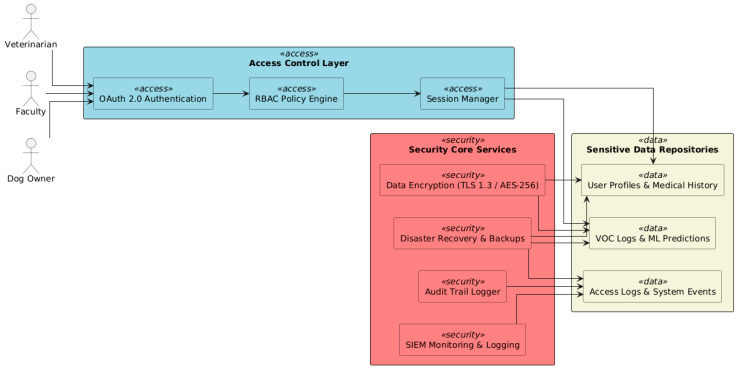
Security and privacy: corresponding with GDPR and the NIS2 Directive compliance requirements.

The system’s design is geared towards maintaining the confidentiality, integrity, and availability (CIA) of the medical and biometric data [22]. The platform adopts a layered security architecture (see Figure 12), which corresponds to GDPR and the NIS2 Directive compliance requirements. By using *data encryption*, all data in transit are encrypted using TLS 1.3, and all data at rest (VOC logs, model predictions, user metadata, etc.) are protected using AES-256. Role-based Access Control (RBAC) facilitates assigning each user (vet, faculty, and owner) a role, with fine-grained permissions to enable access to data and operations. All OAuth 2.0-compliant services manage access tokens and session logs [23]. *Monitor and log* imply that, at a server level, security events and diagnostic activity are monitored through an integrated SIEM solution (Security Information and Event Management) to enable incident detection and forensic traceability of system anomalies. *Audit and backup* represent regular audit trails and daily backups for safety and preparation for disaster recovery long term.

## 7. Interfaces with End-User

Our proposed diagnostic platform caters to several end-user types through secure, role-specific front-end interfaces. A customized dashboard, or portal, tailored to veterinarians, faculty researchers, or dog owners ensures relevant functionality and usability, dependent on the needs and access rights granted to that stakeholder. We utilize web and mobile applications as the user interfaces, connecting them to the backend services via RESTful APIs and securing the connections with role-based access control.

### 7.1. Veterinary Dashboard

**Figure 13:**
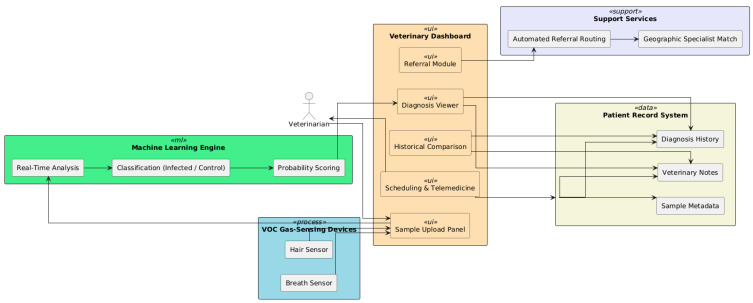
Veterinary dashboard: enables the optimal management of breath and hair sample data, which are obtained using related VOC gas-sensing devices.

In the clinical setting, veterinarians engage with the platform via a purpose-built dashboard that assists with the diagnostic workflow (see Figure 13). This interface enables the optimal management of breath and hair sample data, which are obtained using related VOC gas-sensing devices. Once collected, samples are uploaded through the dashboard, which automatically associates the test with the corresponding patient record, decreasing the administrative burden and facilitating traceability.

An integrated machine learning engine drives the diagnostic system, which analyzes uploaded samples in real-time. After processing, the platform returns interpretable results for veterinarians, in terms of giving an input sample as INFECTED or CONTROL and probability scores. The results are presented via an intuitive interface that highlights the key signal characteristics, trends over time, and potential abnormalities.

In retrospect or representative monitoring, the dashboard empowers veterinarians to compare contemporary samples with historical data. This capability promotes clinical decision-making by allowing for trend analysis and evaluation of treatment efficacy over time. For cases that require additional expertise, including complicated or ambiguous diagnoses, the platform contains a built-in referral mechanism. Through this system, veterinary practitioners can escalate complex or critical cases to regional veterinary hospitals or faculty specialists at veterinary schools. The system automatically routes these referrals based on a set of predefined rules that consider both the geographical proximity of the referral center and the specific medical specialization required for the case. This ensures that each case is directed to the most appropriate expert or facility as efficiently as possible.

Moreover, the dashboard comes with integrated scheduling tools, including both in-clinic and telemedicine appointments. These tools assist veterinary personnel in scheduling patients to visit the clinic or have follow-up appointments, thereby enhancing ongoing care for canine patients.

In summary, the veterinary dashboard serves as the front end for clinical users, streamlining complexity from sample sourcing through to decision support and integration with the broader veterinary landscape.

### 7.2. Academia and Researchers

At the academic and research level, the platform is accessed by academic-sector users through a portal specializing in scientific research and educational use. This interface provides researchers, epidemiologists, and clinicians with more robust capabilities for data exploration, public health monitoring, and collaborative development of diagnostic models.

Access to anonymized datasets of VOC samples collected from veterinary clinics and hospitals is one of the central features of the faculty portal. These datasets are de-identified under GDPR and ethical research guidelines for query and information export for epidemiological studies and machine learning experiments by academic teams. This capability aids diverse research goals—from understanding disease transmission patterns to exploring novel diagnostic hypotheses—and enables researchers in general to extract insights from this growing amount of public genomic sequence data.

**Figure 14:**
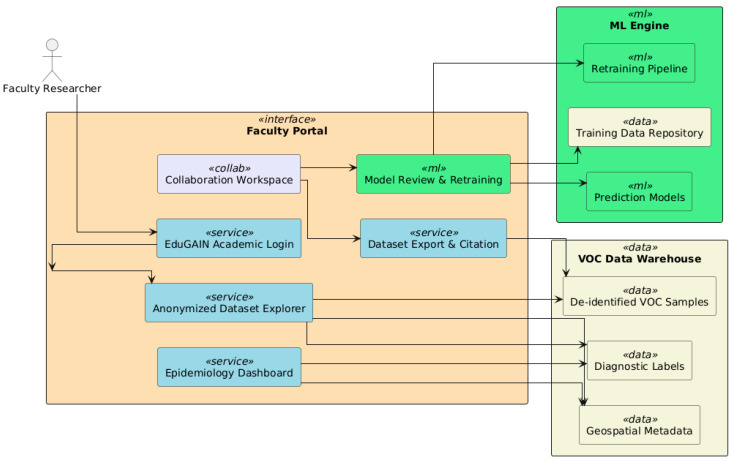
The portal features are used to collate diagnostic results and visualize them in heatmaps, charts, and timelines, reflecting the rates of infection, local prevalence, and OCR/VOC biomarker distributions over time.

Alongside data access, the portal features an epidemiology dashboard (see Figure 14) that is used to collate diagnostic results and visualize them in heatmaps, charts, and timelines reflecting the rates of infection, local prevalence, and OCR/VOC biomarker distributions over time. The dashboard provides real-time insights into the geographic and clinical dynamics of canine leishmaniasis, making it a valuable tool for surveillance and veterinary public health planning.

The platform also creates a feedback loop between researchers and diagnostic models. Faculty can review ML outputs, annotate misclassifications, and refine training data through an integrated retraining interface to improve model performance. This enables continuous learning and system adaptation, bringing the platform in line with open science and reproducible research principles.

Additionally, the portal also connects with academic project management and publication tools. Seamless engagement in multi-institutional research projects is supported through features like dataset citation, institutional login through EduGAIN, and collaborative workspaces. These capabilities enable academic publication, student training, and collaboration across borders for zoonotic disease control.

In conclusion, the faculty interface turns the diagnostic platform from a solely clinical tool into a center for applied veterinary research, knowledge dissemination, and strategic decision-making, expanding its value.

### 7.3. Dog Owner Portal

**Figure 15:**
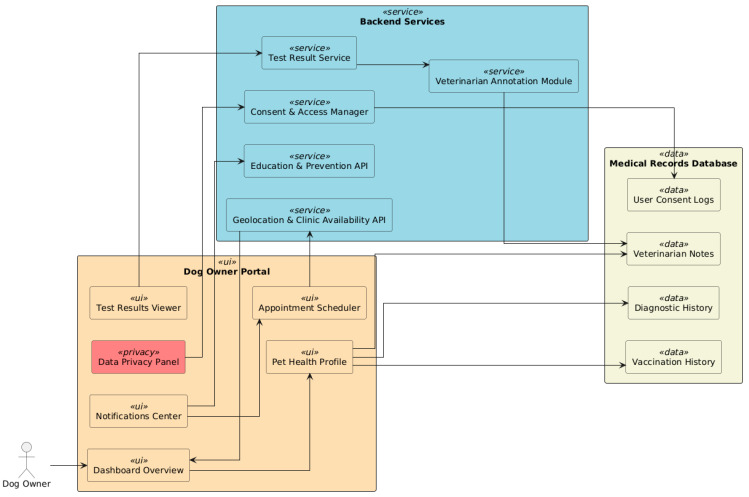
Dog owner portal: pet owners can re-engage with their dogs’ health through communication, scheduling, and result interpretation.

For dog owners, the platform (see Figure 15) is a simple, intuitive application on the web and mobile that makes access to veterinary diagnostics holistic and user-centric. With this interface, pet owners can re-engage with their dogs’ health through communication, scheduling, and result interpretation.

When logging in to the portal, owners are greeted with a dashboard built around their pet’s profile. Each dog registered in the database has a stand-alone health record that captures demographic information, vaccination status, prior diagnostic results, and veterinarian notes. To provide transparency and continuity of care, the system allows users to access historical health data in a time-based manner.

At the core of this portal’s functionality is the appointment scheduling module, which utilizes geolocation services to identify veterinary clinics that are part of the VOC-based leishmaniasis screening program located near the user. The system suggests the best available time slots based on proximity, clinics’ availability, and diagnostic capacity; the owner can then schedule and manage appointments straight from the interface. This significantly lowers the barrier to entry compared to traditional booking systems and guarantees timely diagnostic interaction.

Four hours after a diagnostic session, the test-result viewer shows results in layperson’s terms. Each report, along with an explanatory note from an attending veterinarian, includes the diagnostic classification (e.g., INFECTED or CONTROL). Visual cues like status icons or traffic-light color-coding help pet owners quickly gauge urgency and the next best steps.

For this app, we also developed a notification system that sends real-time alerts and reminders. These consist of vaccination reminders, upcoming appointments, disease prevention recommendations, and educational content based on seasonal outbreaks or parasite risks in the user’s region.

The portal provides owners with complete control over their pet’s data sharing, in line with data privacy and ethical use. In an integrated data privacy panel, users can control consent preferences for academic research and choose which clinics, hospitals, or faculty members can access their dog’s medical information. This facet complies with contemporary data protection standards, discourages abuse, and fosters confidence in the system’s integrity.

The dog owner portal fosters a link between pet owners and the veterinary ecosystem by providing access to medical records, diagnostics, and communication, thereby promoting early disease detection, better compliance with care plans, and a more proactive approach to canine health.

## 8. Implementation Steps

The practicality and performance of the suggested non-invasive diagnostic platform were demonstrated by conducting a proof-of-concept implementation. Implementation and testing of the platform have been performed in Romania—an emerging veterinary concern is present in this part of the world, especially in southern and rural areas with high vector activity.

### 8.1. Deployment Overview

The pilot infrastructure extends its functionalities to and wishes to be linked to a geographically dispersed network of participating veterinary entities, including veterinary clinics with gas-sensing stations for VOC sampling veterinary hospitals/hubs where cases of increased complexity are escalated for advanced diagnostics. Veterinary medicine faculties (at national/international university levels) are responsible for research coordination, epidemiological monitoring, and model validation. The VOCs used for analysis were taken from 72 dogs registered in the system through a dedicated mobile/web portal.

In Tunisia, the eco-epidemiological and clinical exploratory study started with visiting every dwelling in the selected endemic area and investigating the dogs encountered there by using structured questionnaires. Dog owners from the selected dwelling provided information regarding the health and demographics of each enrolled dog. The age of explored dogs is several months to several years. Every dog was subjected to clinical exploration, and blood samples were collected in plain tubes for serum extraction and another blood sample in ethylenediamin tetra-acetic acid (EDTA) tubes for DNA extraction and genomic parasite identification using the loop-mediated isothermal amplification (LAMP) method, which was performed recently. For serodiagnosis, an in-house-developed Enzyme-Linked Immunosorbent Assay (ELISA), using different crude antigens of two species of *Leishmania* parasites (*L. major* and *L. infantum*) present in Tunisia as support, and an indirect immunofluorescence (IFI) test, using *L. infantum* promastigotes as support, were used. Canine leishmaniasis serodiagnosis was also performed using a commercial test, the “ID Screen Leishmaniasis Indirect” ELISA kit (IDvet Innovative Diagnostics, France), which was used as described in its manual. Blood samples were collected and transferred to a container maintained at +4 °C to the IPT laboratory. There, serums were extracted after centrifugation at 3000 rpm and recuperated in sterile tubes, which were then kept at −20 °C until use. For blood samples in ethylenediamin tetra-acetic acid (EDTA) tubes, the DNA was extracted, using a commercial Qiagen kit from the buffy coat (WBC-rich layer), which was obtained after blood centrifugation at 1000 tr/mn. Then, the extracted and purified DNA was conserved at −20 °C until use.

Each clinic/unit was supplied with a complete diagnostic kit comprising the breath and hair sampling devices, sensor calibration materials, and access credentials to the platform. The system’s onboarding involved staff training sessions, ethical consent procedures, and standardization workshops to harmonize sampling protocols across institutions.

### 8.2. Testing the Platform Using the VOCs from 72 Dogs

The platform has been designed to replicate specific use cases and functionalities by utilizing data sourced from 72 dogs. Its primary aim is to identify and analyze the volatile organic compounds (VOCs) emitted, facilitating precise modeling and validation across a range of detection and diagnostic scenarios.

By harnessing the VOC profiles obtained from these 72 canine subjects, the system creates an authentic setting for testing and enhancing its performance. This methodology guarantees a comprehensive simulation of real-life conditions, thereby aiding in the advancement and fine-tuning of detection algorithms as well as associated features.

### 8.3. Notes and Lessons Learned

The deployment yielded a few operational insights. Providing consistent sensor calibration was vital to enable comparable results across locations. Standard operating procedures, along with regular calibration schedules, were key. Additionally, internet connectivity limitations in rural clinics occasionally result in delayed data synchronization. Local coaching and batch uploads helped mitigate this issue. Adoption was shaped heavily by our stakeholder training and onboarding efforts. As a result, the clinics with dedicated digital champions had greater engagement and sample throughput.

Diagnostic capacity will be enhanced for other zoonotic diseases with the integration of federated learning for cross-border model training with an aim for system-wide expansion across other EU member states.

## 9. Case Study and Discussions

### 9.1. Technical and Operational Challenges

The calibration of VOC sensors across multiple geographic sites was one of the most significant technical challenges encountered during the pilot phase. Despite using standardized sensor hardware and calibration protocols in the system, variations in environmental conditions (e.g., temperature, humidity, and background air composition) measurably affected sensor outputs. Diagnostic Accuracy could be affected without regularization and environmental normalization. Moving forward, the platform will announce real-time environmental compensation models and automated self-calibration mechanisms.

The other challenge was ensuring data quality and signal integrity. Some clinics reported occasional noise in the signal or packet loss—from the data sent over the internet —in cases where network conditions were unstable or in rural locations. Many of these concerns were mitigated with local caching strategies, but they reveal the need for a more robust edge-device architecture and asynchronous data pipelines.

Model drift is a less predictable but equally critical problem. Due to seasonality, dietary changes, geographic locations, or other time variables, the VOC profiles may change over time, causing a shift in the feature distributions as the platform continues to learn from new data. Thus, frequent retrievals of representative datasets may be required for retraining and/or federated learning approaches to prevent overfitting.

### 9.2. Scalability and Generalizability to Other Conditions

The qualitative approach of the proposed platform allows for both vertical and horizontal scalability. The cloud-native architecture on which it is built is microservices-based, making the onboarding of new clinics, hospitals, and academic partners possible with minimal configuration. The platform’s VOC analysis capabilities are not limited to leishmaniasis. With the right sensor reconfiguration and AI model retraining, the same infrastructure might be leveraged to diagnose other infectious or metabolic conditions in animals, including respiratory infections, kidney disease, or affective co-infections.

Scalability also raises the question of interoperability with national veterinary systems. In order to support broader adoption, particularly at a governmental or EU level, future iterations of the platform should be interoperable with electronic health record (EHR) standards and data governance frameworks across borders.

### 9.3. Ethical Aspects and Trust in the User

The long-term success of the platform depends on its ethical and social implications. In terms of data privacy, the system follows GDPR principles and specifically offers consent management and data anonymization pipelines, primarily for research purposes. On top of compliance, trust must be earned. For example, transparency about how the algorithm arrived at its decision is critical when veterinarians and pet owners are making clinical decisions based on automated predictions. Although the system provides probabilities and traceable histories of diagnoses, offering interpretability, this does not involve humans in the equation.

The spectral profile is common in signals where useful information lies in gradual trends over time rather than high-frequency variations. Examples include *electrodermal activity* (*EDA*) in physiological monitoring; *environmental sensor data*, such as temperature or humidity changes; and *low-speed mechanical* system vibrations or *background ambient* signals where no strong cyclical interference is present.

Also, ensure that informed consent from dog owners—especially when samples are uploaded for academic purposes—is clearly obtained and can be revoked. There have been positive results from allowing users to manage their data-sharing preferences.

Finally, building veterinarians’ trust is critical. Many clinicians in the pilot were excited about what the platform could do for them, while others raised concerns about AI’s place in clinical decisions. This knowledge gap can be addressed by continual training, the application of human-in-the-loop feedback systems, and joint validation with veterinary faculties.

In conclusion, despite the technical feasibility and acceptance of the proposed platform, there remains a persistent need to focus on calibration, scalability, and ethical implementation if it is to become a robust and trustworthy tool that can enrich and inform the wider veterinary community.

### 9.4. Geographic and Epidemiological Distribution

The implications for the data and modeling approaches are that region-specific calibration and adaptive modeling techniques will be needed to adjust for geospatial variability.

**Figure 16:**
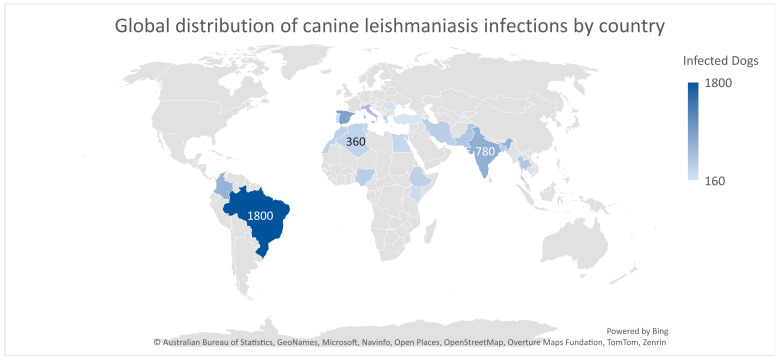
Global distribution of canine leishmaniasis infections by country [37].

To put the regional trends observed in the pilot data into additional context, we provide a global map of the number of canine leishmaniasis infections estimated by country (Figure 16). This figure combines reported cases as well as literature-based estimates from multiple countries and prioritizes areas known to have endemic leishmaniasis infantum transmission.

Note the highest-burden clusters seen in South America and southern Europe on the map, most notably, Brazil, Spain, and Italy—countries with established transmission cycles and need systems to cope with the reported burden. High infection burdens are also reported in India, Colombia, and Iran, reflecting the zoonotic and geographic heterogeneity of the disease. CVL is endemic in the following countries: Brazil, Italy, Morocco, and Tunisia. Reports of disease expansion have been observed in endemic countries and in areas where CVL is not endemic, such as Uruguay [37].

In Africa, Morocco, Algeria, and Ethiopia, moderate case estimates are also reported, which are likely under-reported due to lower veterinary infrastructure or surveillance. Consistently, preliminary evidence from Southeast Asia (e.g., Thailand, Pakistan, and Bangladesh) highlights potential deficiencies in detection and reporting systems.

This worldwide perspective highlights the need for cross-border veterinary disease surveillance and non-invasive diagnostic tools, especially in under-represented regions or where resources are limited.

#### Infection Burden by Country

**Figure 17:**
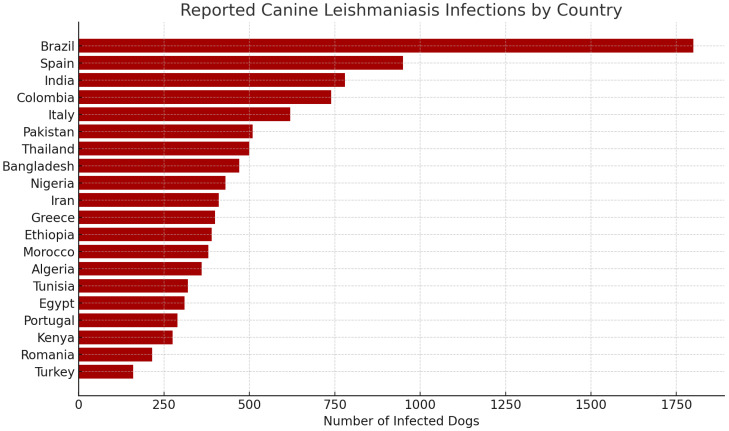
Reported canine leishmaniasis infections by country.

Along with the previous analysis of spatial distribution, Figure 17 provides a horizontal bar chart listing the countries in order by the number of reported canine leishmaniasis infections. This visualization highlights the unequal burden seen in a handful of countries. Brazil has the highest number of infections, as expected, given its endemic status and exposure to sandflies. Also, Spain, India, and Colombia round out the list, reflecting a combination of climate factors, rural pet populations, and active surveillance systems. Notably, countries including Ethiopia, Pakistan, and Bangladesh also have a significant case burden, highlighting an increasing demand for access to diagnostics in developing countries.

This ranking can be beneficial for deploying non-invasive diagnostic technologies in priority areas, such as those developed in our proposed platform.

## 10. Conclusions

We proposed a comprehensive, multi-layered platform for the non-invasive diagnosis of canine leishmaniasis. We discussed the implementation of volatile organic compound (VOC) sensing technologies and the development of machine learning-based classification models alongside a secure, cloud-native software infrastructure.

The proposed platform provides accurate, fast, and accessible disease detection by combining real-time AI predictions, geospatial coordination across veterinary clinics and hospitals, and role-based dashboards for stakeholders. Strong cybersecurity measures, adherence to data privacy standards, and modular architecture make the platform secure, flexible, and scalable.

Results from the pilot deployment validate the utility of the platform in clinical workflows, its acceptability among dog owners, and its research value for academic institutions. The architecture has shown to be robust and adaptable to future extensions, such as diagnosis of other diseases, deployment in the field with mobile sensing units, and integration with federated learning frameworks.

In summary, this research marks a major step towards the digitization of veterinary diagnostics, paving the way for innovations in animal health, disease surveillance, and AI-assisted veterinary care.

## 11. Patents and Copyrights

1. Marius Iulian, Mihailescu. Veterinary Enhanced Telemetry & Analysis for Lab-based Knowledge (VETALK), Medical Veterinary Cloud-based Platform for Chemical Compounds Analysis from Signal Processing, Part 1—Software Architecture and System Design. Registration Certificate no.: 1685/28.04.2025, filled on 20 April 2025 and issued on 28 April 2025 by Romanian Office for Copyright (ORDA).
2. Marius Iulian, Mihailescu. Veterinary Enhanced Telemetry & Analysis for Lab-based Knowledge (VETALK), Medical Veterinary Cloud-based Platform for Chemical Compounds Analysis from Signal Processing, Part 2—Graphic User Interfaces. Registration Certificate no.: 1684/28.04.2025, filled on 20 April 2025 and issued on 28 April 2025 by Romanian Office for Copyright (ORDA).

## Data Availability

Data are contained within the article. The original contributions presented in this study are included in the article.
